# Chronic developmental exposure to traffic-derived PM_2.5_ has limited skeletal effects in mice

**DOI:** 10.1038/s41598-026-54342-1

**Published:** 2026-05-28

**Authors:** Joudi Altaleb, Min Feng, Yushi Zhao, Xing Zhou, Hui Chen, Brian G. Oliver, Ian Mudway, Ulrich Hansen, Richard L. Abel

**Affiliations:** 1 School of Public Health, Faculty of Medicine, Imperial College London, London, United Kingdom; 2 School of Life Sciences, Faculty of Science, University of Technology, Sydney, Australia; 3 Department of Surgery & Cancer, Faculty of Medicine, Imperial College London, London, United Kingdom; 4 Department of Mechanical Engineering, Faculty of Engineering, Imperial College London, London, United Kingdom

**Keywords:** PM2.5, Osteoporosis, Metals, Bone, Traffic pollution, Development, Diseases, Environmental sciences, Medical research, Risk factors

## Abstract

Epidemiological studies link traffic proximity to increased osteoporosis and fracture risk in older adults, but mechanistic relationships remain unclear. Following our recently published hypothesis regarding metal-induced bone fragility, we performed an exploratory study to investigate whether chronic exposures to metals in traffic-derived PM_2.5_ during development compromise bone health in young adult mice. This opportunistic investigation utilised stored biological material from a concurrent sub-chronic exposure study. BALBc mice were divided into PM_2.5_ exposed and control groups over 12 weeks. Starting at 6 weeks of age PM_2.5_ exposed mice received daily intranasal administration of 10 µg/mL filtered particulates collected from Sydney roadside air while controls received saline. Metal accumulation was quantified using ICP-MS and bone structural properties were assessed via micro-CT. Mechanical properties were evaluated through three-point bending tests at 4 and 8 and 12 weeks of exposure. No significant differences were observed between groups across any parameter. Both groups exhibited comparable concentrations of endogenous and exogenous metals at all examined time points. Bone geometric properties and mechanical characteristics including yield stress and ultimate tensile strength and stiffness were similar between groups with normal age-related development in both. These findings suggest that metal accumulation in bone is not significant in early life and is not associated with a deterioration of mechanical properties under the specific conditions of this exploratory model. It is more likely that any adverse effects require a cumulative lifetime burden and age-related decline in protective mechanisms rather than immediate developmental vulnerability. This shifts the focus from early-life susceptibility to long-term risk and establishes a methodological pipeline for future longitudinal research.

**Supplementary Information:**

The online version contains supplementary material available at 10.1038/s41598-026-54342-1.

## Introduction

Osteoporosis, a debilitating skeletal disorder characterised by reduced bone strength and increased fracture risk, represents a significant global health burden. Fragility fractures, a hallmark of osteoporosis, have a devastating impact on health, mortality, and quality of life, affecting 500 million people worldwide^1,2^, causing 37 million fractures annually^1,3^, and costing the global healthcare system over $400 billion per year^4^. Recent epidemiological studies have begun to identify environmental factors that may contribute to this burden.

Growing epidemiological evidence suggests a concerning association between exposure to airborne particulate matter (PM) smaller than 2.5 μm (PM_2.5_) and an increased risk of osteoporosis and osteoporotic fractures^5–8^. For instance, in Beijing, individuals residing within 50 m of high-traffic areas exhibited a 5% lower bone mineral density at the hip and twice the risk of hip fractures compared to those living more than 300 m away^5^. Similarly, studies in the USA and more recently in European cohorts have demonstrated that individuals living near high-traffic areas have a higher risk of bone mineral density loss^5,6,9^. These findings underscore the urgency of investigating the underlying mechanisms driving this observed association^5,6^.

Following our recently published hypothesis regarding traffic-derived metal bioaccumulation in bone^10^, we posited that this adverse correlation could be related to metals associated with airborne particulate matter incorporating into bone tissue and directly exerting toxic effects. These metals, such as lead (Pb), chromium (Cr), nickel (Ni), and manganese (Mn), derived from various traffic sources including exhaust emissions, tyre and brake pad abrasion, and the resuspension of road dust^7^, can substitute for calcium (Ca) in the bone mineral. Replacing Ca with exogenous metals in the bone’s hydroxyapatite crystal structure can weaken the crucial bond between the mineral and collagen, leading to increased bone brittleness and reduced mechanical strength^8,11,12^. Beyond this, in-vitro and in-vivo studies have shown that various metals can also be directly toxic to bone cells, disrupting bone remodelling by increasing osteoclastic bone resorption and decreasing osteoblastic formation, leading to an imbalance that favours bone loss^13–16^.

Beyond the direct effects of metals, it is also likely that the capacity of PM_2.5_ to induce systemic inflammation contributes to the observed bone health deficits. Exposure to PM is known to trigger an inflammatory response throughout the body, and chronic systemic inflammation is a recognised risk factor for bone loss and osteoporosis^17^. This indirect pathway, acting in concert with the direct effects of metal incorporation, may provide a more comprehensive explanation for the epidemiological findings. While these observational studies provide compelling evidence, establishing a direct causal relationship and elucidating the precise mechanisms of metal accumulation in bone, as well as the role of systemic inflammation, requires robust experimental data. Even low, but chronic, exposure to these pollutants could lead to significant long-term bone mass loss due to bioaccumulation and persistent inflammation^17^. Recent in-vivo studies have demonstrated that chronic exposure to traffic-derived PM_2.5_ can indeed cause systemic toxicity, as evidenced by liver pathology in mice^18^.

In this study, our overall aim was to investigate whether early-life exposure to traffic-derived PM_2.5_ compromises bone health in young adult mice, thereby providing a mechanistic foundation for the epidemiologically observed associations between traffic proximity and bone fragility in human populations. We primarily aimed to investigate the potential for translocation and incorporation of metals specifically associated with the ultrafine fraction of airborne particulate matter into bone, focusing on the critical developmental period. We hypothesised that this early life stage may allow for greater translocation of these ultrafine particles due to fast bone cell turnaround and their associated metals to the circulation and subsequently to developing bone. To achieve this, our specific objectives included quantifying metal bioaccumulation patterns in developing bone tissue following PM_2.5_ exposure, assessing the impact of metal exposure on bone structural development using micro-CT analysis, evaluating functional consequences through mechanical testing of bone strength properties, and determining the temporal progression of any effects across a 12 week developmental exposure window. We utilised a nasal instillation model, which, while a compromise compared to inhalation but better for quantifiable delivery, was considered a reasonable approach for a proof-of-principle experiment to assess the potential for metal accumulation in bone. This represents the first examination of metals and metalloids in bone over this crucial developmental window, addressing a critical gap between robust epidemiological evidence linking traffic proximity to bone fragility and the absence of mechanistic data establishing causality, particularly during early development.

We hypothesised that chronic exposure to traffic-derived PM_2.5_ would lead to the incorporation of exogenous metals into developing bone tissue, potentially compromising bone health. Specifically, we predicted that PM_2.5_-exposed mice would exhibit elevated concentrations of exogenous metals in bone tissue compared to controls, with progressive accumulation over the exposure period leading to reduced bone mass and compromised bone strength.

## Method

### Ethics

The mice experiments were approved by the Animal Care and Ethics Committee at the University of Technology Sydney (Approval # ETH21–6236) and performed according to the recommendations for using and caring for experimental animals for scientific purposes issued by the National Health and Medical Research Council of Australia.

The authors have complied with the ARRIVE guidelines. The CT scanning, mechanical testing, and ICP-MS were all performed in a blinded fashion.

### Mouse model

**Fig. 1:**
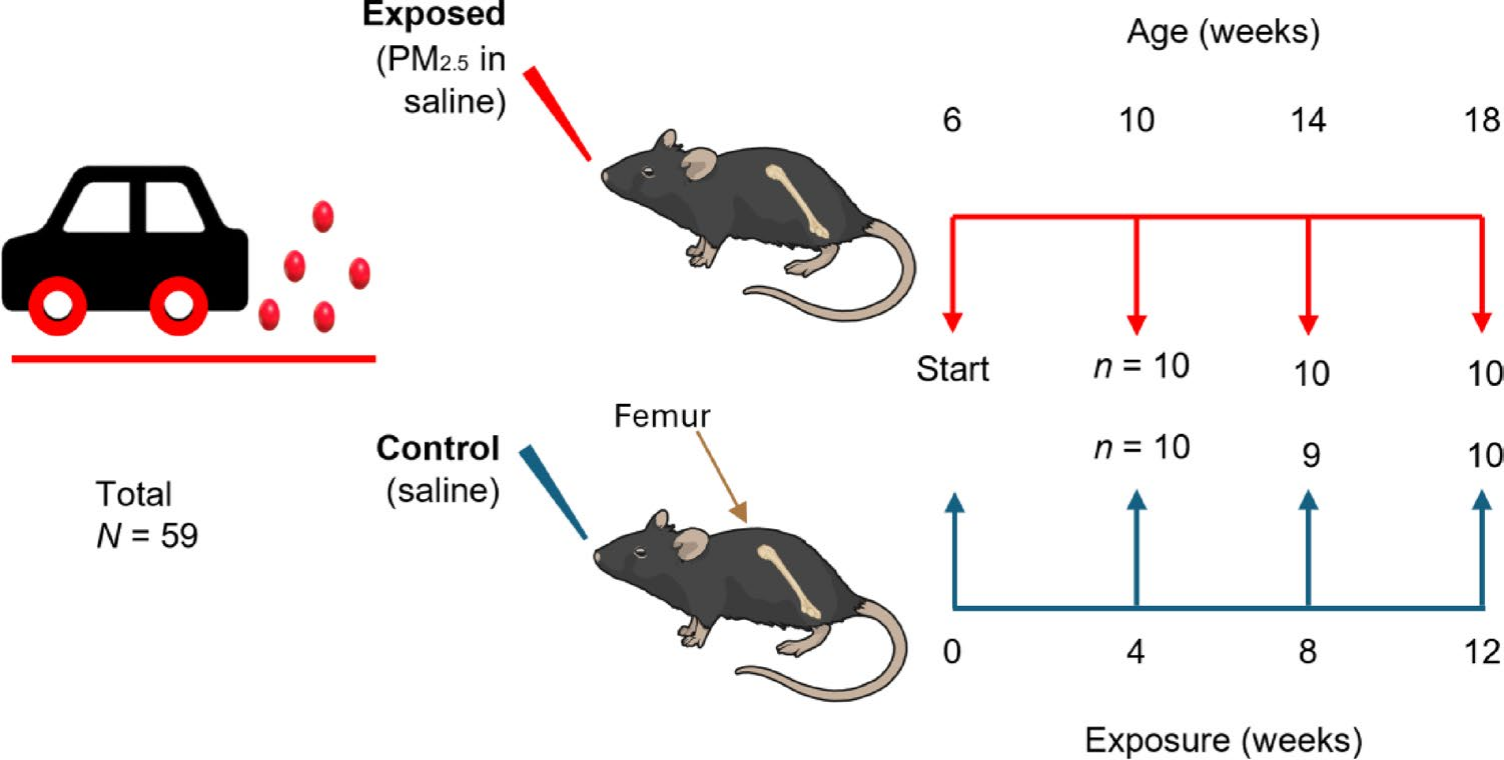
Mouse model of inhalation of traffic-derived PM2.5 collected from a busy road. Exposure started at 6 weeks of age. The mice we exposed to 10 µg of extracted PM2.5 per day.

5-week-old, male BALB/c mice were used in the present study due to their known vulnerability to traffic related pollutants^18–20^ (Fig. 1). The mice were housed at 20 ± 2 °C, and 12 h light:12 h dark cycle and acclimatised for 1 week with ad libitum access to water and standard laboratory rodent chow^18–20^. All the mice used the same water supply. The mice were euthanised with a ketamine/xylazine mixture (90/15 mg/kg, i.p.)^18^. Further details on the model and their housing have previously been described^18^.

The body weight of the mice was measured at the 3 time points (10, 14 and 18 weeks of age). At 10 weeks (*n* = 10), the mean bodyweight (g) ± SEM of the PM_2.5_-exposed group was 21.4 ± 0.25 and that of the control group was 21.6 ± 0.33. At 14 weeks (*n* = 10), the mean bodyweight (g) ± SEM of the PM_2.5_-exposed group was 23.4 ± 0.44 and that of the control group was 22.9 ± 0.30. At 18 weeks (*n* = 10), the mean bodyweight (g) ± SEM of the PM_2.5_-exposed group was 24.1 ± 0.30 and that of the control group was 23.9 ± 0.39.

The mice were exposed to PM_2.5_ collected adjacent to a major road in Sydney (Australia). Ambient PM_2.5_ metal concentrations are presented in Table s1. The mice were randomised and divided into PM_2.5_-exposed (*n* = 29) and control (*n* = 30) groups. Exposure started at 6 weeks of age, and the mice were sacrificed at 10, 14, and 18 weeks of age. The samples were pooled and the entire cohort was administered the same batch of extracted PM_2.5_. The PM_2.5_ exposure was for 4, 8, and 12 weeks. The PM_2.5_ -exposed group received a daily intranasal dose of 10 µg of PM_2.5_ containing filtered metals, calculated based on average PM_2.5_ air pollution levels London (10–20 µg/m³ depending on the proximity to roads) and a (maximum) daily human exposure of (290 µg). We have published the non-metal compositions in our previous study on lung pathology^23^, which include chloride, nitrite, nitrate, sulphate, sodium, ammonium, potassium, organic carbon, and elemental carbon.

The total volume of air breathed by a mouse over 24 h is 278 times lower than in a human^20^. Therefore, the equivalent mouse exposure dose is 10ug/day. We used 10ug/day to account for the convention of using approximately 10 times higher doses of agonists in mouse models, and to account for losses due to the route of administration (up to 50%). The control group received intranasal saline only (40 µg). The PM_2.5_ exposed group received daily intranasal dose of 10 µg of PM_2.5_ containing filtered metals. PM_2.5_ was suspended in 40ul saline for intranasal delivery, sham treatment was 40ul of saline.

### Analysis of traffic derived metal pollution

Four PM_2.5_ filter samples were collected using JH-120 F Automatic Medium Flow PM Samplers from roadside air on a high-traffic road in Sydney, Australia during July-August 2022 to characterise the metal composition of traffic-derived particulate matter used in the exposure protocol. The PM_2.5_ was collected using on polytetrafluoroethylene membranes with a pore size of 3.0 μm (ProSciTech) and extracted using 90% methanol and sonicated in a water bath as explained in^19,20^. Filter samples were analysed by Eurofins Environment Testing using standardised heavy metal analysis methods (Method M7) with detection limits ranging from 0.1 to 5 µg per filter depending on the analyte. The analysis revealed that Zinc (Zn) was the predominant traffic-derived metal, consistently detected across all samples at concentrations ranging from 5.0 to 14 µg per filter, with peak concentrations observed in mid-August. Copper (Cu) was detected in three of four samples (1.3–2.2 µg per filter) and Pb was present in two samples (1.1–1.4 µg per filter), both metals consistent with vehicular sources including brake pad wear and residual fuel additives. Arsenic (As), Cadmium (Cd), Chromium (Cr), and Nickel (Ni) concentrations were below detection limits (< 0.1–1 µg per filter) in all samples. This metal profile confirmed that the collected PM_2.5_ contained measurable concentrations of traffic-derived metals, validating the environmental relevance of the exposure model, with Zn representing the primary metal exposure followed by Cu and Pb in order of abundance.

### Sample preparation

The lower limbs of mice were carefully dissected and debrided using a ceramic scalpel to prevent metal contamination and to ensure precise separation of muscle from bone. Femurs were immediately collected and stored in saline to maintain hydration and structural integrity. All dissected femurs were then stored at − 80 °C until required for mechanical testing or digestion for metal/metalloid analysis. 59 femora were effectively prepared for analysis; one femur was damaged prior to dissection (14-week control group). The samples were thawed in room temperature saline bath to prevent structural microdamage. The limb in the best condition was chosen for analysis, and the left and right limbs were not distinguished.

### Micro-CT scanning

Micro-CT imaging was performed using a Zeiss Versa 510 scanner to obtain high-resolution images of the femurs. Prior to scanning, each femur was wrapped in parafilm to prevent dehydration and placed into a 3D-printed cylindrical tube for secure positioning. To optimise scanning efficiency and minimise processing time, two samples were scanned simultaneously in opposite orientations. The scan setting were X-ray energy (kV) 90.00, power (W) 8.00, 1601 projections. Smoothing = 0.5, Ring Removal = None, Beam hardening = 5%, Binning = 2.

The voxels were anisotropic. In‑plane pixel dimensions (X and Y) were equal to each other, but the voxel depth (Z) was different due to binning = 2 during reconstruction (to accelerate this exploratory pilot). The voxel sizes range from 8 to 9 μm. The reported range of 8–9 μm refers the variation in absolute voxel size between scans caused by small differences in sample‑to‑detector distance; it does not describe the anisotropy itself. No phantom calibration was used because our outcome was direct mechanical strength.

We measured cortical cross-sectional area (CSA) as it is a good measure of growth and deposition as CSA increases with age^21^. Since bone length differs between the three age groups, CSA was normalised by diaphyseal length (CSA/Length)^21^. Diaphyseal length itself was measured to test for the effect of PM_2.5_ exposure on longitudinal growth^21^. Due to the fragility of the proximal and distal femora, many samples were missing these regions; therefore, diaphyseal length was used instead of whole‑bone length. Finally, in the absence of trabecular bone, we measured cortical bone volume within the diaphysis and normalised it by length as another measure of growth and deposition, since volume also increases with age.

Following scanning, CT image sequences were imported into ImageJ for further processing. Images were cropped to isolate individual femora, and brightness and contrast were adjusted to ensure clear differentiation between the femur and the background. Cortical bone was segmented by global thresholding with a lower threshold of 27,295 and an upper threshold of 65,353 on a 16-bit grayscale (0–65,535). The images were imported into Mimics software. The femora were resliced to achieve a vertical orientation for standardised measurements.

Geometric measurements including cross-sectional area (CSA), diaphyseal length, cortical bone volume, cortical bone thickness, and second moment of area (I) were obtained as follows. Diaphyseal length was determined by counting the number of slices constituting the femoral diaphysis and multiplying that number by voxel depth. Cortical bone volume was calculated by multiplying the number of white (bone) voxels by the volume of a single voxel. CSA, second moment of area (I), and cortical bone thickness were measured at 50% of diaphyseal length using the Slice Geometry tool in the BoneJ plugin within ImageJ. All measurements were performed consistently by three independent analysts to ensure reproducibility and accuracy across all femur samples.

### Mechanical testing

Mechanical properties of the femora were tested by 3-point bending using a CellScale Univert (20 N load cell, accuracy 0.04 N). The load was applied at a rate of 5 mm/min until the failure of the femora. A force displacement reading was taken every 0.1 s. The third trochanter were consistently placed downwards to test in the mediolateral (ML) direction according to established protocol of testing mouse femora in the ML direction^22^. Mediolateral direction 3-point bending was chosen because the femur shows the highest strength in this direction and is naturally how mouse bone bears the highest loading^22^.

**Fig. 2:**
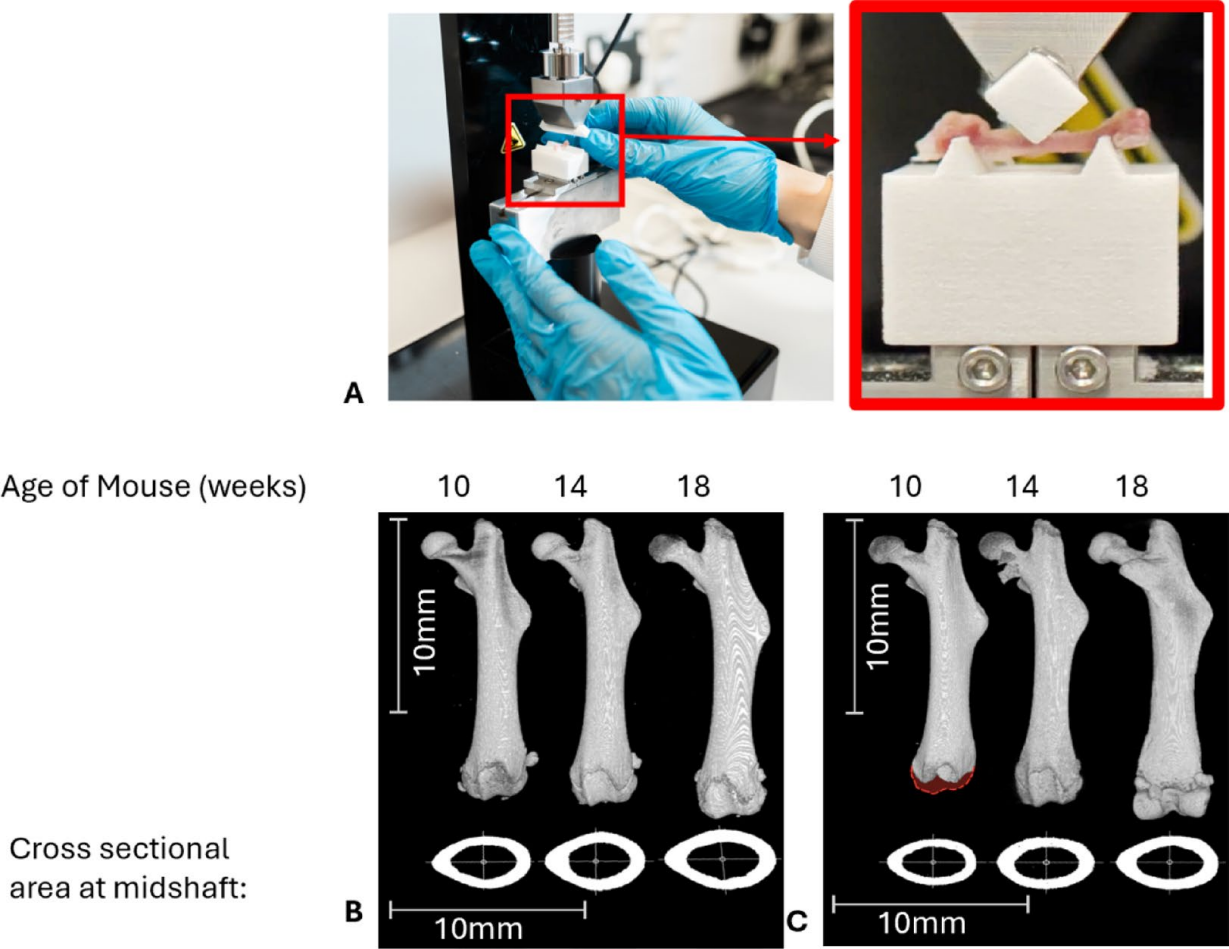
(**A**) Three-point bending test setup for mechanical evaluation of mouse femora. (**B**) CT-scanned images and cross-sectional areas (CSA) at the midshaft of three median femora in good condition from three age groups treated with PM2.5. (**C**) CT-scanned images and CSA of three median femora from three age groups treated with Saline. The red area a drawn approximation of the distal femur.

The span length was 9.64 mm. The ratio of span length to bone width is 6 which is lower than what beam theory assumes which is > 16:1^21^. The ratio of span length to bone width for most mice bone 3-point bending tests are around 5, so our span length is acceptable for this application^21^. This span length selection is inevitable because of how mouse femora is shaped. The machine was calibrated with the biomaterials testing application to find the start point. Each femur was mechanically loaded to fracture (Fig. 2).

The 3D printed stands were made of EOS Store PA 2200—Polyamide 12 with a tensile modulus of 1650 MPa. This was a necessary compromise as the 3D printed stands to avoid metal contamination of the femora. We quantified fixture compliance by compressive testing of the stand and loading bar without specimens across the load range used in bending. The measured load–displacement resulted in an average fixture compliance $\:{c}_{fix}\approx\:0.0102\:mm/N$. All bone displacements were corrected as $\:{\delta\:}_{corr}={\delta\:}_{meas}-\:{c}_{fix}F$ where $\:{\delta\:}_{corr}$ is the corrected displacement, $\:{\delta\:}_{meas}$ is the measured displacement, and F is the force at measures displacement.

Output was a force-displacement graph. Using the line tool on BoneJ, both the inner and outer diameter (do and di) of the midshaft of the femur (50% of diaphysis length), distance from the centroid of the cross section to the outermost point on the cross section (c) were measured. The moment of inertia about the bending axis, I, c, do and di values were noted and were used alongside 3-point bending readings to determine Yield Stress, Ultimate Tensile Stress, Stiffness and Young’s Modulus (E). The equations for converting the force displacement graphs into the young’s modulus, ultimate tensile stress, yield stress can be found in Eqs. 1 and 2^21^. The F refers to either yield force for the calculations of yield stress or the maximum force for the calculation of ultimate tensile stress.

1 $$\:\sigma\:=Flc4I$$

2 $$\:E=F{L}^{3}d48I$$

### ICP-MS

ICP-MS analysis was performed on acid digested femur samples to quantify metal concentrations following 4, 8, and 12 weeks of exposure at the London Metallomics Facility. Samples were transferred into pre weighed trace grade 15 ml centrifuge tubes and then dried in the oven overnight at 70 °C. Samples were then allowed to cool and weighed to measure the dry tissue weight. Samples were digested by adding 0.5 mL of Optima grade concentrated HNO_3_ (67–69% w/w; Fisher Scientific) and place into Oven at 60 °C to digest over a 1-hour period. The digested samples were diluted to 10 mL using purified water with a resistivity ≥ 18.2 MΩ cm from an Milli-Q IQ 7015 ultrapure and pure water purification system. As part of the dilution, samples were doped with the internal standard Rh using a high purity standards 100 mgL^− 1^ standard solution to obtain sample concentrations of 10 µgL^− 1^.

All measurements were conducted on a Perkin Elmer NexION 5000 Inductively Coupled Plasma Quadrupole Mass Spectrometer (ICP-QQQMS) under Dynamic Reaction Cell (DRC) mode at the London Metallomics Facility, King’s College London. The following elements were quantified, the selected isotope and DRC analysis mode is indicated: ^27^Al (Kinetic energy discrimination (KED)), ^31^P (O_2_), ^40^Ca (Ammonia), ^48^Ti (O_2_), 50 V (Ammonia), ^52^Cr (Ammonia), ^55^Mn (Ammonia), ^56^Fe (Ammonia), ^59^Co (Ammonia), ^60^Ni (Ammonia), ^63^Cu (Ammonia), ^66^Zn (Ammonia), ^75^As (O2), ^95^Mo (O2), ^111^Cd (KED), ^120^Sn (KED), ^121^Sb (Ammonia), ^138^Ba (O_2_), ^195^Pt (KED), ^202^Hg (KED), ^208^Pb (Ammonia). The introduction system to the instrument was a Cetac ASX-560 autosampler coupled to a SeaSpray glass nebulizer fitted to a quartz cyclonic spray chamber. Argon plasma flow and nebulizer gas flow rates were 18 Lmin^− 1^ and 0.98 Lmin^− 1^, respectively.

Quality control (QC) of ICP-MS measurements was ensured through repeat measurements of acid blanks, a 15 µgL^− 1^. Quality control standard made from using a 100 mgL^− 1^ High Purity Standards multi-element standard and a 10-fold dilution of the CRM-TMDW from High Purity Standards. Analyte measurements were normalized to the internal standard Rh to account for instrument drift and matrix effects, and measurements were subsequently blank corrected by removing the average analyte intensity of repeat blank measurements. The corrected analyte intensity was converted to concentration measurements using the linear equation generated by the calibration curve of the standards.

The blanks are run numerous times to generate data for Limit of Detection (LOD) and blank subtraction calculations. CRM and QCs were run every 20 samples to ensure accuracy of the measurement throughout the run. Each ICP-MS sample is measured using 5 replicates conducted by the instrument, in terms of physical replicates there were 59 femur samples which are biological replicates.

### Data analysis

**Fig. 3:**
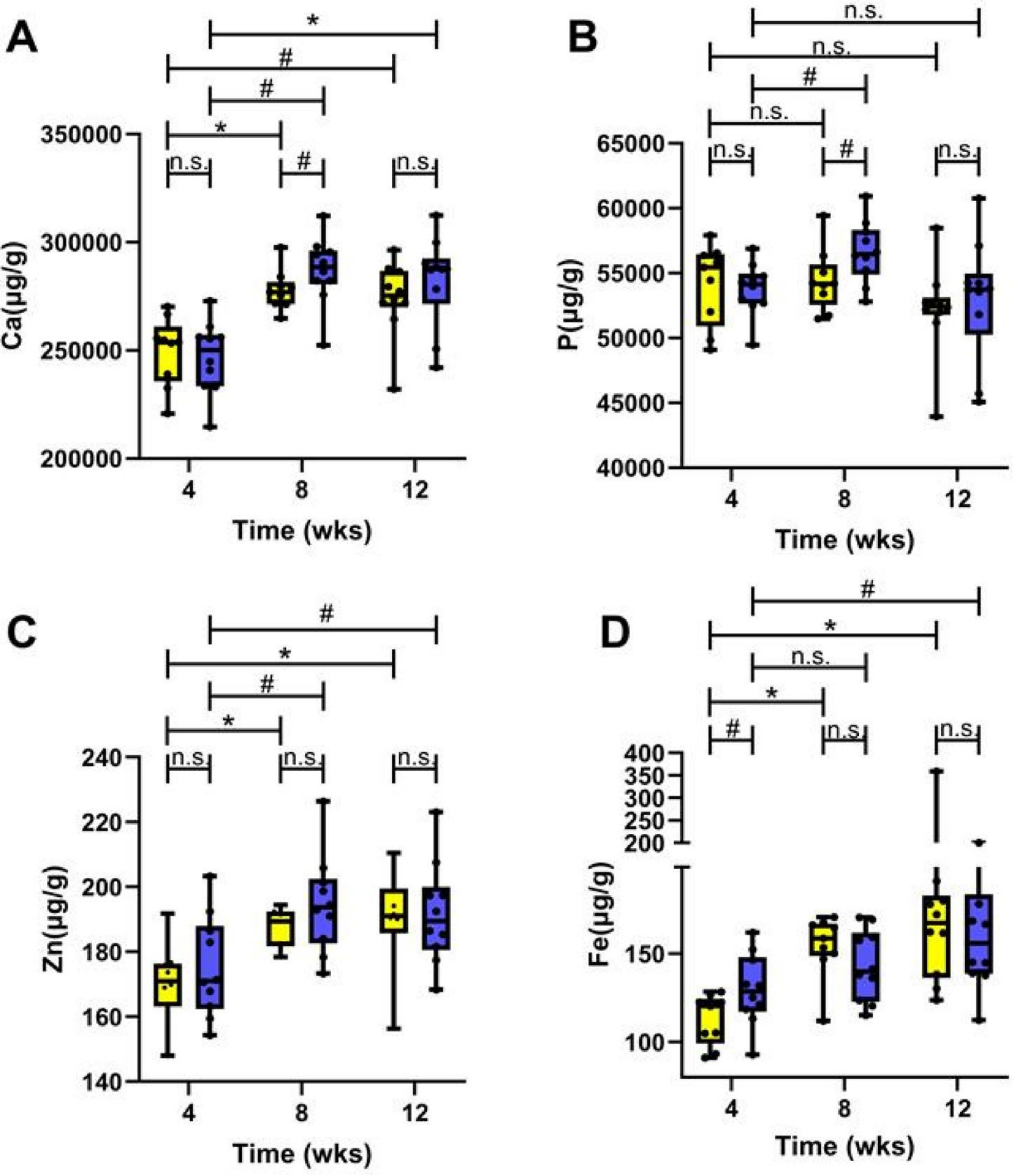
Mouse Femoral concentrations of endogenous metals at 4, 8, and 12 weeks in control and PM_2.5_ exposed groups: (**a**) Ca, (**b**) P, (**c**) Zn, (**d**) Fe. The blue colour bars are the experimental group, and the yellow colour bars are the control group. Significance is denoted as * for *p* < 0.05, ** for *p* < 0.01, # for *p* < 0.10, and n.s. for *p* > 0.10.

**Fig. 4:**
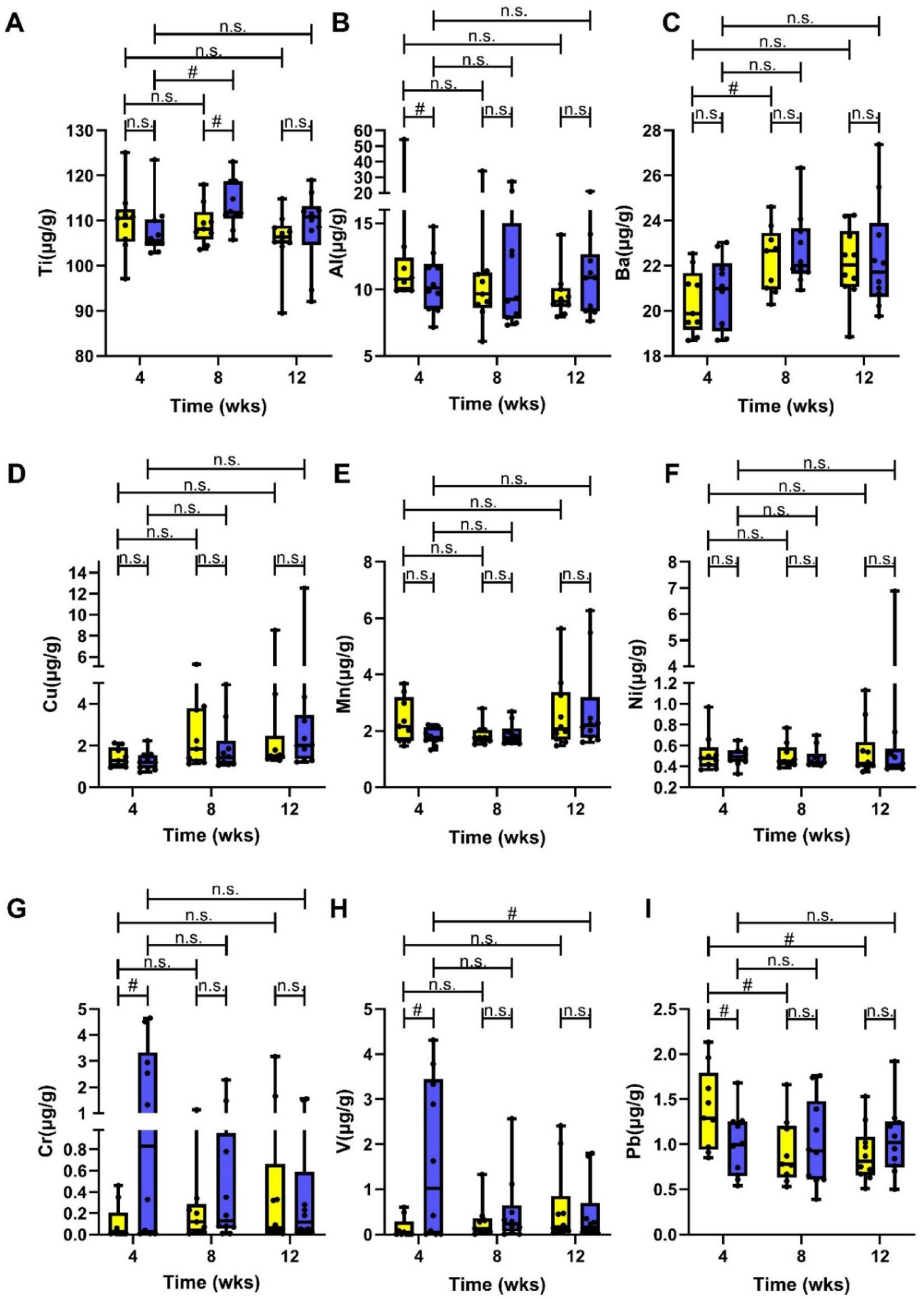
Mouse Femoral concentrations of exogenous metals at 4, 8, and 12 weeks in control and PM_2.5_ exposed groups: (**a**) Ti, (**b**) Al, (**c**) Ba, (**d**) Cu, (**e**) Mn, (**f**) Ni, (**g**) Cr, (**h**) V, (**i**) Pb. The blue colour bars are the experimental group, and the yellow colour bars are the control group. Significance is denoted as * for *p* < 0.05, ** for *p* < 0.01, # for *p* < 0.10, and n.s. for *p* > 0.10.

**Fig. 5:**
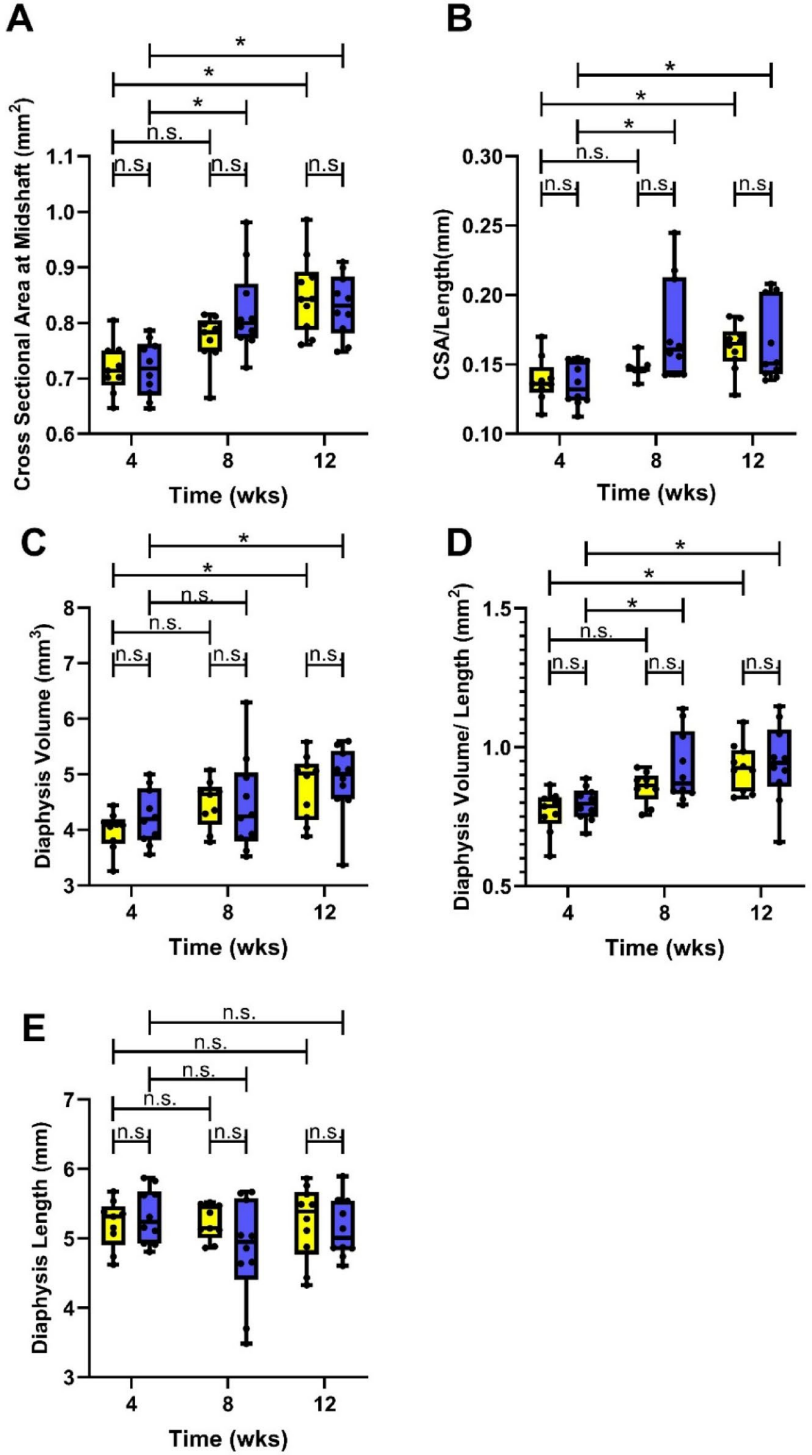
Morphological changes in femoral bone across 4, 8, and 12 Weeks of control or PM_2.5_ challenges: (**a**) Cross-sectional area, (**b**) Ratio of cross-sectional area to length, (**c**) Diaphysis volume, (**d**) Ratio of diaphysis volume to length, (**e**) Length of mouse femora diaphysis. The blue colour bars are the experimental group, and the yellow colour bars are the control group. Significance is denoted as * for *p* < 0.05, ** for *p* < 0.01, # for *p* < 0.10, and n.s. for *p* > 0.10.

**Fig. 6:**
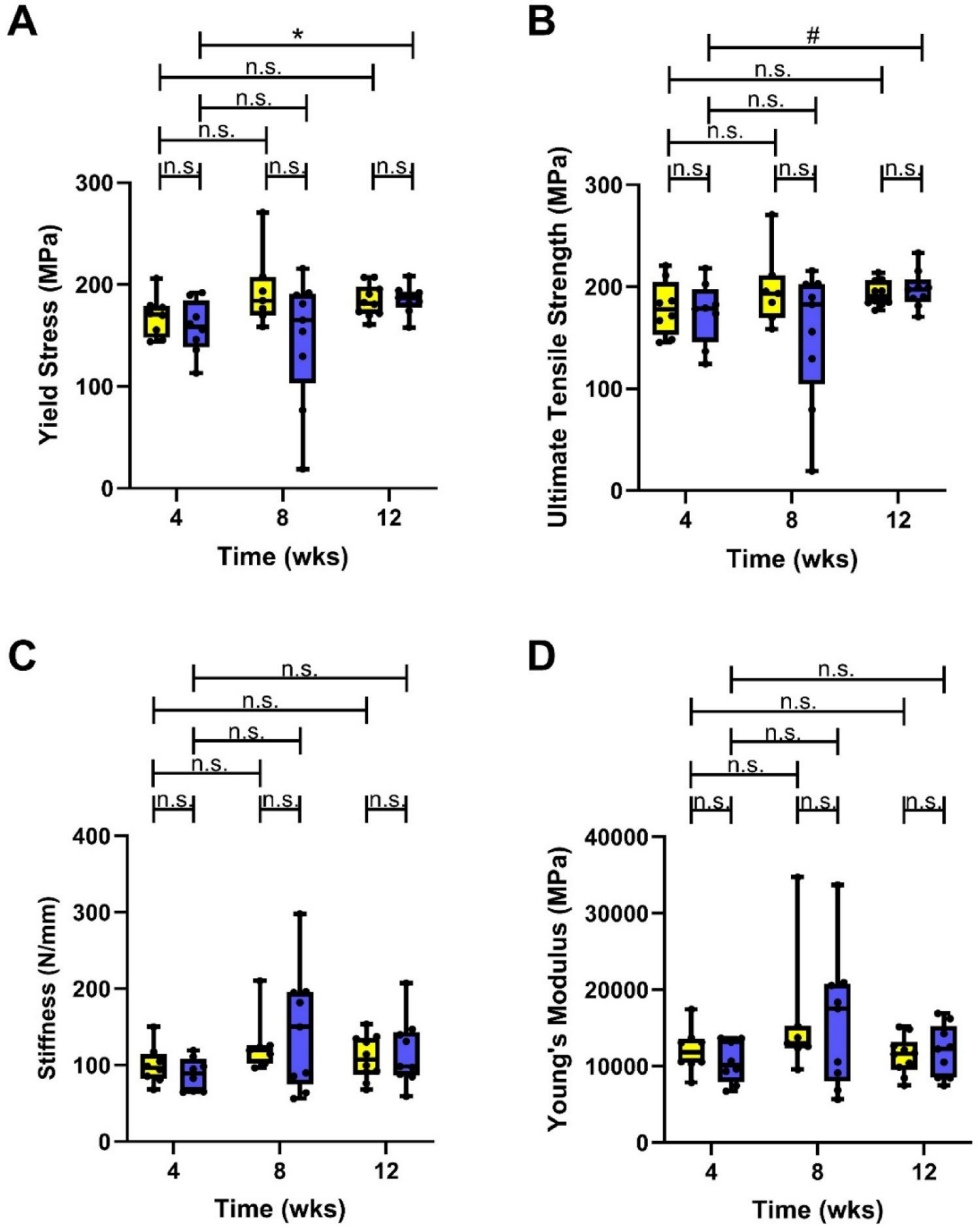
Changes in the mechanical properties of femoral bone after 4, 8, and 12 weeks of control or PM_2.5_ challenge: (**a**) Yield Stress, (**b**) Ultimate Tensile Stress (UTS), (**c**) Stiffness, (**d**) Young’s Modulus. The blue colour bars are the experimental group, and the yellow colour bars are the control group. Significance is denoted as * for *p* < 0.05, ** for *p* < 0.01, # for *p* < 0.10, and n.s. for *p* > 0.10.

Nonparametric statistics were applied because the sample size per group/stage was small (9–10) and some were non-normally distributed. PM_2.5_-exposed and control groups characterised by calculating the median and IQR. Statistical comparisons were made using the Kruskal–Wallis test, with post hoc testing between groups using the Mann-Whitney U test. Data in Figs. 3, 4, 5 and 6 are illustrated as both individual data points and summarised as box plots, with median concentrations, 25th and 75th percentiles and the 95% confidence interval. The blue bars represent the exposed groups, and the yellow bars represent the control groups. Statistical comparison between groups was performed using the Mann-Whitney U test.

Associations between the measure elements and the mechanical variables were performed using the Spearman rank correlation. Factor analysis was conducted using Principal Component Analysis (PCA) with Varimax rotation to identify significant factor loadings. Specifically, variables with factor loadings greater than 1 were considered significant. All data was analysed using GraphPad Prism (v10.3.1) and IBM© SPPS© Statistics (version 26).

## Results

Bone elemental composition data, expressed per dry weight, are grouped as either being predominately ‘endogenous’; related to bone mineral (Ca and P), or as dominant biometals (Zn or Fe) (Fig. 3), or as ‘exogenous’, if there is a basis for believing their concentration in bone may be more reflective of environmental uptake (Ti, Al, Ba, Cu, Mn, Ni, Cr, V and Pb) (Fig. 4).

### Endogenous metal concentrations

PM_2.5_-exposed and control mice exhibited a time dependent accumulation of Ca in bone between 4 and 8 weeks, plateauing between 8 and 12 weeks (Fig. 3a). Note, these are experimental dates corresponding to 10, 14, 18 weeks of age. No statistical differences were noted between the control and PM challenged mice at any of the examined time points, though there was a trend (*p* = 0.07) suggesting an increase in Ca in the PM challenge group at 8 weeks. Phosphorus (P) was more consistent over time, but like Ca there was evidence for a trend (*p* = 0.07) toward increased concentrations in the PM challenged versus controls at 8 weeks (Fig. 3b). Zn and Fe concentrations also demonstrated accumulation in bone, particularly evidence between 4 and 8 weeks (Fig. 3 panels c and d respectively), with no differences in concentration between the PM treated and control groups. It is important to note that the increase in Ca, P, Fe, and Zn is also due to the general increase in the bone mass of mice due to growth into adulthood. Full data are provided in supplementary Table s2.

### Exogenous metal concentrations

All nine exogenous metals were detected in both PM_2.5_-exposed and control groups (Fig. 4a–i). In contrast to the endogenous metals, with measurement of the exogenous metals, we saw very little evidence for a time dependent accumulation, except for Barium (Ba) (panel c) where there was some evidence supporting an increase at 8 weeks, in the control animals. Overall, there were no statistically significant differences in metal concentrations between control and PM treated mice at any of the examined time points, but a few trends were observed: an increase in Titanium (Ti) in the PM treated animals at 8 weeks (*p* = 0.05, Fig. 4, panel a); increases in Cr (*p* = 0.09, Fig. 4, panel g) and Vanadium (V) (*p* = 0.08, Fig. 4, panel h) in the PM challenged mice at 4 weeks, and a decrease of bone Pb in the PM treated animals (*p* = 0.07, Fig. 4 panel i) at 4 weeks. Full data are provided in supplementary Table s3.

Overall across all animals (*n* = 58) we saw significant association between bone Ca concentrations with P (ρ = 0.57, *p* < 0.001), Ti (ρ = 0.66, *p* < 0.001), Fe (ρ = 0.38, *p* < 0.01), Cu (ρ = 0.28, *p* < 0.05), Zn (ρ = 0.72, *p* < 0.001) and Ba (ρ = 0.69, *p* < 0.001), Supplemental Fig. s1. Of the exogenous metals examined significant associations were observed between V with Cr (ρ = 0.96, *p* < 0.001) and Ni (ρ = 0.41, *p* < 0.01). When a Principal Component Analysis was performed it identified 5 elemental clusters, the first dominated by P, Ca, Ti, Zn and Ba; the second by V and Cr; the third, Mn and Ni; Fourth, Cu and Zn, and fifth, Cu and Pb, Supplemental Fig. s1. The correlation and clustering of bone metals in the control and PM treated animals are shown separately in Supplemental Fig. s2. Overall, the general patterns of associations were similar to those seen in the full analysis.

### Bone geometry and mechanics

In both PM_2.5_ exposed, and control groups bone geometry increased progressively from 10 to 18 weeks of age, experimental weeks 4–12 (Fig. 5) for all geometric parameters but length. No significant differences were noted between the control and PM-treated animals at any time point (See Fig. 5). Full data are provided in supplementary Table s4. Bone mechanical properties remained constant in PM_2.5_-exposed and control animals from 4 to 12 weeks of exposure (Fig. 6), with the only significant change being an increase in yield stress from 4 to 12 weeks for the PM_2.5_ exposed group from 4 to 12 weeks. PM_2.5_-exposed and control groups exhibited similar bone mechanics at all stages of development (See Fig. 6). Full data are provided in supplementary Table s5.

Overall, no significant associations were noted between bone elemental concentration and mechanical properties when examined across all samples, saline and PM-treated animal combined (See Supplemental Fig. s3). When the data was analysed separately by treatment group, some evidence of decreased mechanical strength was evident in the PM-treated group, with bone Mn (stiffness and Young’s modulus) and Ni (UTS and stiffness).

## Discussion

This study investigated the effects of chronic PM_2.5_ metal exposure on bone health in young adult mice by analysing metal accumulation and geometric properties and mechanical performance following 4 and 8 and 12 weeks of daily intranasal administration. The research was designed as a first preliminary examination of the hypothesis that traffic-related metal emissions drive bone fragility^10^. It addresses epidemiological findings that demonstrate associations between proximity to high-traffic areas and increased risk of osteoporosis in human populations^5^.

While the current study utilised young adult mice in contrast to the older human populations examined in many epidemiological studies this age difference is itself informative. By focusing on bone development during a period of rapid growth the mouse model enables investigation of the innate protective mechanisms of bone tissue when exposed to environmental metals. Thus, the mouse study provides a controlled framework to probe the earliest biological interactions between traffic-related metals and bone. It addresses an important gap in our understanding of how environmental exposures might initiate subtle changes that only manifest as fragility in later decades. Comprehensive analysis revealed no significant differences between PM_2.5_ exposed and control groups across any measured parameter. These findings suggest that the detrimental effects of metal exposure observed in some cohorts may not manifest immediately during the developmental period but rather may require longer exposure durations.

### Metal accumulation

The absence of differential metal accumulation indicates that the PM_2.5_ exposure did not result in measurable metal burden differences in bone tissue. This occurred despite the challenge being sufficient to produce significant systemic effects including liver toxicity in the same animals^18^. ICP-MS analyses used the whole bone, so it is not clear whether metal preferentially accumulated in the trabecular or cortical bone. The detection of metals including Ti and Aluminium (Al) and V and Ni and Cr in both groups suggests ubiquitous environmental background within the laboratory setting or dietary intake.

Table s6 shows the list of metals found in the animal chow. This demonstrates that the animals will have received a significant proportion of their metal intake from the diet. However, all the mice received the same diet, we would have predicted that we would still be able to observe an increment in the animal receiving metal via their respiratory system.

The stability of exogenous metal concentrations across the exposure period, in contrast to the expected age-related increases in endogenous metals, suggests that developing bone possesses effective buffering systems that prevent metal accumulation, even when systemically absorbed PM_2.5_ particles are causing damage in other tissues. These findings highlight that bone tissue exhibits unique resistance to metal burden during the developmental period, fundamentally different from the vulnerability observed in metabolically active organs.

While we found no clear evidence of excess metal accumulation we did observe underlying associations within the bone elemental profile. Most notably all animals showed a significant correlation between Cr and V. These elements are established tracers for fuel oil combustion^24^. The exploratory factor analysis identified these elements as a distinct cluster separate from the endogenous bone mineral. Although not statistically significant the elevated concentrations observed after four weeks of treatment suggest a potential external source. Recent evidence from Scheepers et al. (2025) suggests that bone mineral density in children is specifically associated with PM_2.5_ absorbance and NO_2_ rather than total particulate mass. This population-level coherence suggests that the combustion-derived fraction of traffic pollution warrants greater attention than coarse mode components.

### Bone geometry and mechanics

The absence of significant differences in bone geometry and mechanical performance directly reflects the comparable metal accumulation patterns. These findings suggest that young actively growing bone tissue possesses sufficient protective capacity to maintain structural properties during the developmental window. We did observe some suggestive evidence that metal accumulation after the challenges was associated with a weakening of bone, but this underlying association did not manifest as concentration differences at any examined time point.

### Biological mechanisms and species differences

The apparent resistance of young murine bone likely stems from robust protective mechanisms. Rapid appositional growth continuously deposits new uncontaminated bone matrix on the periosteal surface while resorbing existing bone on the endosteal surface^25,26^. This process provides a unique protective mechanism absent in mature bone where metal accumulation cannot be similarly eliminated through coordinated deposition and resorption^27^. Furthermore, murine skeletons contain proportionally more dense cortical bone which may be less sensitive to environmental factors than the trabecular bone that predominates in human vertebral bodies^27^. It is also important to note that mice do not exhibit the same intracortical remodelling processes seen in humans which may further explain the lack of mechanical response in the cortical midshaft.

### Methodological limitations and future directions

The apparent absence of bone effects does not reflect experimental failure as validated by concurrent liver toxicity studies^18^. This study was an opportunistic and exploratory investigation utilising biological material from a collaborator. Consequently, we were restricted by the original experimental design including the number of animals and the specific sacrifice points which were not originally optimised for a bone study. This led to challenges in obtaining consistent trabecular measurements using standard ASBMR metrics.

Additionally quantifying ultra-trace levels of elements such as Cr and V represents a significant analytical challenge. These measurements push the analytical envelope for ICP-MS as concentrations approach the limit of quantification. The high variability observed in these specific metals likely reflects both the proximity to the limit of reporting and natural individual differences in metal clearance. Future studies should include longitudinal designs and aged animal models with naturally diminished bone repair capacity to better model elderly human vulnerability.

## Conclusion

This exploratory investigation demonstrates that chronic PM_2.5_ metal exposure during the developmental period does not compromise bone structural integrity or mechanical properties in young mice. Importantly concurrent studies demonstrated clear hepatic toxicity confirming that the PM_2.5_ challenge was biologically active while bone tissue remained unaffected^18^. These findings reconcile the apparent contradiction between widespread environmental exposure and the observation of bone health effects primarily in older populations. This study fundamentally shifts the temporal framework for understanding metal toxicity from immediate developmental vulnerability to long-term cumulative risk. While young bone tissue exhibits resistance during the critical growth phase the underlying tracer trends are coherent with human epidemiological studies^9^. This validates our opportunistic model as a foundation for identifying high-risk pollutants and established a methodological pipeline for future research into the hypothesis of traffic-derived metal bioaccumulation^10^.

## Supplementary Information

Below is the link to the electronic supplementary material.

Supplementary Material 1

## Data Availability

All data generated or analysed during this study are included in this published article.
