# Supplementary material for: Chronic developmental exposure to traffic-derived PM2.5 has limited skeletal effects in mice
Source: Sci Rep. 2026 May 28;16:24401. doi: 10.1038/s41598-026-54342-1 (PMC13447856; doi:10.1038/s41598-026-54342-1)
Supplement: Supplementary file 1 [file 41598_2026_54342_MOESM1_ESM.docx]

# Supplementary Figures

**Figure s1.** Correlations and clustering of elements in murine bone across all time points and treatments.

**Figure s2.** Correlations and clustering of elements in murine bone following control (saline) and PM treatments.

**Figure s3.** Correlations between mechanical parameters and bone elemental content by treatment group.

# Supplementary Tables

**Table s1.** Metal content of ambient PM2.5 collected in at four time points in July - August 2022.

**Table s2.** The statistical analysis of the endogenous metals found in the mouse femora across three age groups (10 weeks, 14 weeks, and 18 weeks) PM_2.5_ exposed vs control groups. It includes the Median, IQR, median difference, median % difference, and the p-value.

**Table s3.** The statistical analysis of the exogenous metals found in the mouse femora across three age groups (10 weeks, 14 weeks, and 18 weeks) PM_2.5_ exposed vs control groups. It includes the Median, IQR, median difference, median % difference, and the p-value.

**Table s4**. The statistical analysis of the geometric properties of the mouse femora across three age groups (10 weeks, 14 weeks, and 18 weeks) PM_2.5_ exposed vs control groups. It includes the Median, IQR, median difference, median % difference, and the p-value.

**Table s5.** The statistical analysis of the mechanical properties of the mouse femora across three age groups (10 weeks, 14 weeks, and 18 weeks) PM_2.5_-exposed vs control groups. It includes the Median, IQR, median difference, median % difference, and the p-value.

**Table s6.**  The statistical evaluation of endogenous metal levels in mouse femora at 10, 14, and 18 weeks of age. The table reports median percentage difference in red and p-values for pairwise comparisons in green (10 vs. 14, 14 vs. 18, and 10 vs. 18 weeks) in both exposed and control groups

**Table s7.** The statistical evaluation of exogenous metal levels in mouse femora at 10, 14, and 18 weeks of age. The table reports median percentage difference in red and p-values for pairwise comparisons in green (10 vs. 14, 14 vs. 18, and 10 vs. 18 weeks) in both exposed and control groups.

**Table s8.** The statistical evaluation of geometric properties of mouse femora at 10, 14, and 18 weeks of age. The table reports median percentage difference in red and p-values for pairwise comparisons in green (10 vs. 14, 14 vs. 18, and 10 vs. 18 weeks) in both exposed and control groups.

**Table s9.** The statistical evaluation of mechanical properties of mouse femora at 10, 14, and 18 weeks of age. The table reports median percentage difference in red and p-values for pairwise comparisons in green (10 vs. 14, 14 vs. 18, and 10 vs. 18 weeks) in both exposed and control groups.

**Figure s1:** Correlations and clustering of elements in murine bone across all time points and treatments (n=59). Panel A shows the correlation matrix (Spearman rank order) for the elements in bone. The spearman rho is given with the level of significance (*p<0.05. **p<0.01, ***p<0.001). The results of the Principal Component Analysis (PCA) for all time points and treatments are illustrated in panel B. The Scree plot is presented in B*i*, with the contribution of each of the elements to the components in the inset table B*ii*.

**Figure s2**: Correlations and clustering of elements in murine bone following control (saline) and PM treatments. Analyses have been performed on data across the full 10-18-week data sets. Panel A shows the correlation matrix (Spearman rank order) for the elements in bone after saline treatment, in the upper triangle (red text, n=28), and following PM, lower triangle (Blue text, n=30). The spearman rho is given with the level of significance (*p<0.05. **p<0.01, ***p<0.001). Where significant correlations have been seen between the same elements in the saline control and PM treated samples these are highlighted in yellow. Where significant associations are only apparent in one of the treatment groups these are highlighted in grey. The results of the Principal Component Analysis (PCA) for Saline and PM treated animals are illustrated in panels B and C respectively. Scree plots are presented in B*i* and C*i*, with the contribution of each of the elements to the components in the inset tables B*ii* and C*ii*.

**Figure s3**: Correlations between mechanical parameters and bone elemental content by treatment group. Analyses have been performed on data across the full 10-18-week data sets. Panels A and B shows the correlation matrix (Spearman rank order) between the measured mechanical properties and bone elemental content in the saline (n=25) and PM-treated (n=26) animals respectively. Tables show the Spearman rho (ρ) correlation coefficient, with the level of significance: *P<0.05, **P<0.01. YM = Young’s Modulus.

**Table s1.** Metal content of ambient PM_2.5_ collected in at four time points in July - August 2022. The limit of reporting column describes the minimum amount of the element that can be detected in (µg). The metals were collected at 4 time points: July 17^th^, July 27^th^, August 5^th^, and August 16^th^,

| **Element** | **Limit of Reporting**  **(µg)** | **Sample Matrix: Filter Paper (µg)** | | | |
| --- | --- | --- | --- | --- | --- |
|  |  | July 17^th^ | July 27^th^ | August 5^th^ | August 16^th^ |
| As | 1 | <1 | <1 | <1 | <1 |
| Cd | 0.1 | <0.1 | <0.1 | <0.1 | <0.1 |
| Cr | 1 | <1 | <1 | <1 | <1 |
| Cu | 1 | 2.2 | 1.3 | <1 | 1.7 |
| Pb | 1 | 1.4 | <1 | <1 | 1.1 |
| Ni | 1 | <1 | <1 | <1 | <1 |
| Zn | 5 | 5.9 | 5.0 | 6.8 | 14 |

**Table s2.** The statistical analysis of the endogenous metals found in the mouse femora across three age groups (10 weeks, 14 weeks, and 18 weeks) PM_2.5_ exposed vs control groups. It includes the Median, IQR, median difference, median % difference, and the p-value.

| **Variable** | **Age (Weeks)** | **Group** | **Median**  **(μg/g)** | **IQR**  **(μg/g)** | **Median Difference**  **(μg/g)** | **% Median Difference** | **p-value** |
| --- | --- | --- | --- | --- | --- | --- | --- |
| **Ca** | **10** | **Control** | 253858 | 25377 | -3695.00 | -1.46 | 0.97 |
|  |  | **Exposed** | 250163 | 23882 |  |  |  |
|  | **14** | **Control** | 276947 | 10241 | 11652.00 | 4.21 | 0.07 |
|  |  | **Exposed** | 288599 | 15587 |  |  |  |
|  | **18** | **Control** | 275369 | 16795 | 12258.00 | 4.45 | 0.12 |
|  |  | **Exposed** | 287627 | 21194 |  |  |  |
| **P** | **10** | **Control** | 55452 | 5553 | -1313 | 2.37 | 0.55 |
|  |  | **Exposed** | 54139 | 2340 |  |  |  |
|  | **14** | **Control** | 54181 | 3124 | 2297 | 4.24 | 0.07 |
|  |  | **Exposed** | 56478 | 3446 |  |  |  |
|  | **18** | **Control** | 52426 | 1323 | 1316 | 2.51 | 0.28 |
|  |  | **Exposed** | 53742 | 4692 |  |  |  |
| **Zn** | **10** | **Control** | 171.00 | 13.10 | 0.00 | 0.00 | 0.72 |
|  |  | **Exposed** | 171.00 | 25.70 |  |  |  |
|  | **14** | **Control** | 189.40 | 10.80 | 4.30 | 2.27 | 0.182 |
|  |  | **Exposed** | 193.70 | 19.90 |  |  |  |
|  | **18** | **Control** | 191.10 | 13.70 | -1.60 | -0.84 | 0.85 |
|  |  | **Exposed** | 189.50 | 19.40 |  |  |  |
| **Fe** | **10** | **Control** | 120.30 | 25.48 | 8.30 | 6.90 | 0.10 |
|  |  | **Exposed** | 128.60 | 30.70 |  |  |  |
|  | **14** | **Control** | 158.70 | 18.40 | -18.90 | -11.91 | 0.28 |
|  |  | **Exposed** | 139.80 | 39.10 |  |  |  |
|  | **18** | **Control** | 167.30 | 46.60 | -11.40 | -6.81 | 0.80 |
|  |  | **Exposed** | 155.90 | 45.00 |  |  |  |

**Table s3:** The statistical analysis of the exogenous metals found in the mouse femora across three age groups (10 weeks, 14 weeks, and 18 weeks) PM_2.5_ exposed vs control groups. It includes the Median, IQR, median difference, median % difference, and the p-value.

| **Variable** | **Age (Weeks)** | **Group** | **Median**  **(μg/g)** | **IQR**  **(μg/g)** | **Median Difference**  **(μg/g)** | **% Median Difference** | **p-value** |
| --- | --- | --- | --- | --- | --- | --- | --- |
| **Ti** | **10** | **Control** | 110.50 | 7.30 | -5.00 | -4.52 | 0.32 |
|  |  | **Exposed** | 105.50 | 5.80 |  |  |  |
|  | **14** | **Control** | 108.10 | 6.20 | 3.70 | 3.42 | 0.05 |
|  |  | **Exposed** | 111.80 | 8.30 |  |  |  |
|  | **18** | **Control** | 106.40 | 4.00 | 4.40 | 4.14 | 0.11 |
|  |  | **Exposed** | 110.80 | 8.70 |  |  |  |
| **Al** | **10** | **Control** | 10.8 | 2.47 | -0.67 | -6.20 | 0.10 |
|  |  | **Exposed** | 10.13 | 3.41 |  |  |  |
|  | **14** | **Control** | 9.67 | 2.67 | -0.44 | -4.5 | 0.28 |
|  |  | **Exposed** | 9.24 | 7.19 |  |  |  |
|  | **18** | **Control** | 9.12 | 1.44 | 1.78 | 19.52 | 0.80 |
|  |  | **Exposed** | 10.90 | 4.30 |  |  |  |
| **Ba** | **10** | **Control** | 19.88 | 2.53 | 1.10 | 5.53 | 0.84 |
|  |  | **Exposed** | 20.98 | 3.01 |  |  |  |
|  | **14** | **Control** | 22.66 | 2.53 | -0.65 | -2.87 | 0.59 |
|  |  | **Exposed** | 22.01 | 2.02 |  |  |  |
|  | **18** | **Control** | 22.04 | 2.47 | -0.33 | -1.50 | 0.78 |
|  |  | **Exposed** | 21.71 | 3.26 |  |  |  |
| **Cu** | **10** | **Control** | 1.28 | 0.92 | -0.08 | -5.86 | 0.33 |
|  |  | **Exposed** | 1.21 | 0.65 |  |  |  |
|  | **14** | **Control** | 1.85 | 2.58 | -0.41 | -22.16 | 0.36 |
|  |  | **Exposed** | 1.44 | 1.12 |  |  |  |
|  | **18** | **Control** | 1.50 | 1.09 | 0.52 | 34.67 | 0.44 |
|  |  | **Exposed** | 2.02 | 2.11 |  |  |  |
| **Mn** | **10** | **Control** | 2.16 | 1.54 | -0.39 | -17.82 | 0.20 |
|  |  | **Exposed** | 1.78 | 0.53 |  |  |  |
|  | **14** | **Control** | 1.77 | 0.45 | -0.01 | -0.56 | 0.99 |
|  |  | **Exposed** | 1.76 | 0.52 |  |  |  |
|  | **18** | **Control** | 2.07 | 1.69 | 0.13 | 6.28 | 0.85 |
|  |  | **Exposed** | 2.20 | 1.46 |  |  |  |
| **Ni** | **10** | **Control** | 0.48 | 0.19 | 0.02 | 3.13 | 0.80 |
|  |  | **Exposed** | 0.50 | 0.11 |  |  |  |
|  | **14** | **Control** | 0.45 | 0.17 | -0.01 | -2.22 | 0.89 |
|  |  | **Exposed** | 0.44 | 0.11 |  |  |  |
|  | **18** | **Control** | 0.43 | 0.23 | -0.02 | -3.49 | 0.57 |
|  |  | **Exposed** | 0.42 | 0.19 |  |  |  |
| **Cr** | **10** | **Control** | 0.01 | 0.20 | 0.82 | 8200.00 | 0.09 |
|  |  | **Exposed** | 0.83 | 3.33 |  |  |  |
|  | **14** | **Control** | 0.12 | 0.26 | 0.01 | 8.33 | 0.62 |
|  |  | **Exposed** | 0.13 | 0.90 |  |  |  |
|  | **18** | **Control** | 0.07 | 0.64 | 0.05 | 76.92 | 0.87 |
|  |  | **Exposed** | 0.12 | 0.56 |  |  |  |
| **V** | **10** | **Control** | 0.01 | 0.28 | 1.02 | 10150.00 | 0.08 |
|  |  | **Exposed** | 1.03 | 3.43 |  |  |  |
|  | **14** | **Control** | 0.13 | 0.31 | 0.11 | 80.77 | 0.60 |
|  |  | **Exposed** | 0.24 | 0.59 |  |  |  |
|  | **18** | **Control** | 0.17 | 0.78 | -0.01 | -5.88 | 0.59 |
|  |  | **Exposed** | 0.16 | 0.64 |  |  |  |
| **Pb** | **10** | **Control** | 1.29 | 0.85 | -0.30 | -22.87 | 0.07 |
|  |  | **Exposed** | 1.00 | 0.61 |  |  |  |
|  | **14** | **Control** | 0.78 | 0.58 | 0.15 | 18.59 | 0.70 |
|  |  | **Exposed** | 0.93 | 0.87 |  |  |  |
|  | **18** | **Control** | 0.81 | 0.42 | 0.21 | 25.93 | 0.39 |
|  |  | **Exposed** | 1.02 | 0.51 |  |  |  |

**Table s4**: The statistical analysis of the geometric properties of the mouse femora across three age groups (10 weeks, 14 weeks, and 18 weeks) PM_2.5_ exposed vs control groups. It includes the Median, IQR, median difference, median % difference, and the p-value.

| **Variable** | **Age (weeks)** | **Group** | **Median**  **(mm^2^)** | **IQR**  **(mm^2^)** | **Median Difference**  **(mm^2^)** | **% Median Difference** | **p-value** |
| --- | --- | --- | --- | --- | --- | --- | --- |
| **CSA** | **10** | **Control** | 0.72 | 0.08 | 0.01 | 0.02 | 0.90 |
|  |  | **Exposed** | 0.73 | 0.08 |  |  |  |
|  | **14** | **Control** | 0.78 | 0.06 | 0.01 | 0.01 | 0.21 |
|  |  | **Exposed** | 0.79 | 0.07 |  |  |  |
|  | **18** | **Control** | 0.85 | 0.11 | -0.01 | -0.01 | 0.63 |
|  |  | **Exposed** | 0.84 | 0.12 |  |  |  |
|  |  |  | **Median**  **(mm)** | **IQR**  **(mm)** | **Median Difference**  **(mm)** |  |  |
| **CSA/Length** | **10** | **Control** | 0.13 | 0.03 | 0.00 | 0.03 | 0.60 |
|  |  | **Exposed** | 0.13 | 0.01 |  |  |  |
|  | **14** | **Control** | 0.13 | 0.01 | 0.01 | 0.05 | 0.13 |
|  |  | **Exposed** | 0.14 | 0.31 |  |  |  |
|  | **18** | **Control** | 0.16 | 0.03 | -0.01 | -0.09 | 0.63 |
|  |  | **Exposed** | 0.14 | 0.03 |  |  |  |
|  |  |  | **Median**  **(mm^3^)** | **IQR**  **(mm^3^)** | **Median Difference**  **(mm^3^)** |  |  |
| **Volume** | **10** | **Control** | 4.46 | 0.34 | 0.26 | 0.06 | 0.55 |
|  |  | **Exposed** | 4.71 | 0.58 |  |  |  |
|  | **14** | **Control** | 4.96 | 0.93 | 0.17 | 0.03 | 0.58 |
|  |  | **Exposed** | 5.14 | 1.25 |  |  |  |
|  | **18** | **Control** | 5.09 | 0.61 | 0.15 | 0.03 | 0.55 |
|  |  | **Exposed** | 5.25 | 0.89 |  |  |  |
|  |  |  | **Median**  **(mm^2^)** | **IQR**  **(mm^2^)** | **Median Difference**  **(mm^2^)** |  |  |
| **Volume/**  **Length** | **10** | **Control** | 0.80 | 0.10 | 0.00 | 0.00 | 0.55 |
|  |  | **Exposed** | 0.79 | 0.10 |  |  |  |
|  | **14** | **Control** | 0.88 | 0.07 | 0.02 | 0.03 | 0.55 |
|  |  | **Exposed** | 0.91 | 0.18 |  |  |  |
|  | **18** | **Control** | 0.95 | 0.07 | 0.00 | 0.00 | 0.80 |
|  |  | **Exposed** | 0.95 | 0.16 |  |  |  |
|  |  |  | **Median**  **(mm)** | **IQR**  **(mm)** | **Median Difference**  **(mm)** |  | |
| **Length (mm)** | **10** | **Control** | 5.40 | 0.64 | 0.42 | 0.08 | 0.66 |
|  |  | **Exposed** | 5.82 | 0.39 |  |  |  |
|  | **14** | **Control** | 5.78 | 0.49 | 0.00 | 0.00 | 0.28 |
|  |  | **Exposed** | 5.79 | 0.74 |  |  |  |
|  | **18** | **Control** | 5.39 | 0.50 | 0.11 | 0.02 | 0.63 |
|  |  | **Exposed** | 5.51 | 0.47 |  |  |  |

**Table s5:** The statistical analysis of the mechanical properties of the mouse femora across three age groups (10 weeks, 14 weeks, and 18 weeks) PM_2.5_-exposed vs control groups. It includes the Median, IQR, median difference, median % difference, and the p-value.

**Table s6.** A table of the elements found in the animal chow that were also found in the animal bones.

| Element | Element Content |
| --- | --- |
| Ca | 10.1g/kg |
| P | 7.7g/kg |
| Fe | 97.0 mg/kg |
| Cu | 10.6 mg/kg |
| Mn | 87.4 mg/kg |
| Zn | 48.1 mg/kg |
